# Psychometric properties of the Lithuanian version of the functionality appreciation scale

**DOI:** 10.1038/s41598-025-03349-1

**Published:** 2025-05-23

**Authors:** Migle Baceviciene, Vaiva Balciuniene, Rasa Jankauskiene

**Affiliations:** 1 Department of Physical and Social Education, Lithuanian Sports University, Sporto 6, Kaunas, 44221 Lithuania; 2 Institute of Sport Science and Innovations, Lithuanian Sports University, Sporto 6, Kaunas, 44221 Lithuania

**Keywords:** Positive body image, Body functionality, Appreciation, Psychometrics, Factor structure, Lithuanian, Psychology, Human behaviour

## Abstract

This study aimed to assess the psychometric properties of the Lithuanian translation of the Functionality Appreciation Scale (FAS). A sample (*n* = 759) of adults (424 women and 335 men; mean age 23.2 ± 7.1 and age range 18–44 years) participated in the cross-sectional study. Body functionality appreciation, body appreciation, self-esteem, disordered eating and physical activity habits were assessed. Exploratory factor analysis (EFA; *n* = 379) identified a unidimensional factor structure of FAS, supported by findings in a second split-subsample using confirmatory factor analysis (CFA, *n* = 380). FAS scores were invariant across gender groups. No mean score differences were observed between genders. Composite reliability of FAS was established in women (ω = 0.94, 95% CI = 0.92, 0.95) and men (ω = 0.92, 95% CI = 0.90, 0.94). Positive associations of FAS with body appreciation, self-esteem and physical activity habits and negative relationships with disordered eating and BMI (in women) confirmed construct validity. FAS predicted self-esteem over and above the variance accounted for by other study measures. The Lithuanian version of FAS is a psychometrically sound measure of body functionality appreciation. FAS might contribute to a comprehensive assessment of positive body image and can be used in various interventions.

**Supplementary Information:**

The online version contains supplementary material available at 10.1038/s41598-025-03349-1.

## Introduction

Body image is a mental representation of body appearance and its’ functions; it reflects how people perceive, what they think, feel and how they behave concerning their physical appearance and body functionality^1,2^. Many scientific studies have shown that poor body image is related to mental health issues (higher depression and anxiety), more negative health-related behaviours (including dysfunctional eating and exercise) and lower quality of life^3–6^. Although more research has been conducted on negative body image, there has been a recent increase in research based on the concept of positive body image (PBI). PBI is considered a distinct and broad construct which is not the opposite pole of negative body image (most often assessed as body dissatisfaction). PBI includes body appreciation, body acceptance and love, investment in body care, a broad conceptualization of beauty, inner positivity and the ability to reject socio-cultural pressures on appearance^7^. A recent systematic review and meta-analysis showed that body appreciation (a key feature of positive body image) is positively associated with several adaptive well-being constructs (e.g. self-esteem, self-compassion, sexual satisfaction) and is inversely related to several indices of eating and body image disturbances and general psychopathology (depression, anxiety)^8^.

Research on PBI has expanded rapidly in recent decades, with the search for new research tools to comprehensively explore PBI and its links to psychological and physical well-being^7^. Body appreciation is one of the most researched facets of PBI^8^. However, the investigation of other dimensions of positive body image, such as body functionality, is of great importance^7^. Body functionality reflects everything that the body can do or is capable of doing including internal processes, physical capacities, body senses, creative endeavours, communication and self-care behaviours^9,10^. Body functionality is not a facet of PBI; however, body functionality becomes a body image facet when it considers individuals’ perceptions, thoughts, emotions, and behaviours related to their body functions^9,10^. Body functionality appreciation reflects the appreciation, respect, and honour of the body for what it can do. Functionality appreciation includes not only one’s perception of body functionality but also highlights gratitude for it^9–11^.

Functionality appreciation and body appreciation are closely related constructs within the domain of positive body image^11^. While specific correlation coefficients can vary across different studies, existing research typically indicates a positive relationship between the two^12–17^.

Studies also showed that it is constantly associated with lower levels of disordered eating, lower body mass index (BMI) and better mental health and well-being^11^. Specifically, negative significant associations between functionality appreciation and BMI were reported in a samples of Spanish, Italian, Lebanese adults^13,17^, in Romanian and Colombian women^15^. However, no significant correlations were observed in samples with possibly lower BMI variation, such as Japanese^18,19^ and Malaysian adults^16^. The associations between body functionality appreciation and BMI are complex. The Acceptance Model of Intuitive Eating^20^ postulates that when individuals believe that their bodies are unconditionally accepted by significant others, they develop a holistic appreciation of their bodies based on an appreciation of body functionality rather than physical appearance. As a result of this appreciation, individuals are more likely to develop body appreciation, which leads to trusting body cues and eating more intuitively (adaptive eating behaviour involving the attribution of importance to the body’s physiological signals, including the recognition of hunger and satiety). Results of a recent study showed that intuitive eating reduces overeating frequency and counteracts maladaptive eating behaviors^21^. Therefore, body functionality appreciation is related to more healthy BMI.

According to sociocultural models of disordered eating^22,23^, the associations between pressures to meet idealised body standards and disordered eating are mediated by internalisation of stereotyped beauty ideals, body comparisons, body dissatisfaction (and/or body objectification). The appreciation of body functionality may assist in prioritising body functionality over body appearance, thereby more effectively resisting sociocultural pressures. Consequently, this may result in the development of a more positive body image and a resistance to disordered eating. Previous research has indicated negative associations between appreciation of body functionality and disordered eating^13–15,17^. A significant prospective study also found that appreciation of body functionality negatively predicted symptoms of eating disorders^24^.

It has been hypothesised that appreciation of body functionality may facilitate engagement in health-promoting behaviors, such as mindful physical activity^25,26^. Previous research has demonstrated that participation in sports (especially competitive) is associated with a more functional body image, as individuals engaged in exercise often prioritize physical performance over appearance^27^. The direction of attentional focus during physical activity is a central component of mindfulness, which has been shown to positively influence positive body image^28^. Specifically, mindful body acceptance, reduced body surveillance (a dimension of self-objectification) and pleasantness during exercise have been linked to higher levels of body appreciation during physical activity^29^. Increased enjoyment and satisfaction during exercise are key predictors of intrinsic motivation and long-term adherence to physical activity^30,31^. Conversely, lower perceived body functionality has been associated with heightened self-objectification^9^. The results of initial cross-sectional studies showed that athletes report higher functionality appreciation than non-athletes^32,33^. In summary, it is plausible to expect that people who value the functionality of their bodies will be more likely to engage in activities that promote well-being, such as prolonged physical activity^9^.

Body functionality appreciation or being grateful for the body as a process has been constantly related to higher general self-esteem^10,13–16,34^. Self-esteem is defined as a positive or negative attitude towards oneself and can also be described as an individual’s sense of self-worth^35^. Individuals with higher self-esteem tend to exhibit greater confidence and independence, lessening their reliance on external validation for self-esteem. Appreciating body functionality fosters an internal sense of worth and effectiveness^36^. This shift in mindset promotes more stable and authentic self-esteem, which is less dependent on external validation.

Given its clear psychological relevance, functionality appreciation represents a promising, yet underexplored, construct within health psychology and behavior research. Focusing on assessing functionality appreciation is significant because it provides the possibility to have a more comprehensive picture of the PBI and its role in the development of a healthy lifestyle^11^. It is therefore essential to have reliable and internationally valid questionnaires to measure body functionality appreciation.

To assess this construct, Alleva and colleagues^10^ developed the 7-item Functional Appreciation Scale (FAS). By performing both exploratory and confirmatory factor analyses (EFA and CFA) with adult samples from the United States, a unidimensional factor structure of FAS was determined. In addition, adequate internal consistency and three weeks of test–retest reliability for the original FAS scale were obtained. Moreover, FAS scores were invariant across men and women, and had adequate construct (convergent, criterion-related, divergent) and incremental validity. For example, the FAS adequate convergent, criterion-related, divergent validity was approved through significant associations with scores on body surveillance, body appreciation (body image measures), depressive symptomatology, self-esteem, life satisfaction (psychological well-being measures), and self-compassion (positive self-care measure). The FAS’s adequate incremental validity was confirmed through the significant prediction of psychological well-being, where FAS scores predicted indicators of psychological well-being over and above scores on other measurements of body image^10^.

The FAS has been increasingly employed by researchers for use in a diverse range of national contexts. Its good psychometric properties with unidimensional factor structure have been globally demonstrated in adults from a community in the United Kingdom^37^, Italy^13^, the Republic of Cyprus^14^, Poland^38^, Romania^15^, Japan^19^, Malaysia^16^, Lebanon^39^, China^40^, Colombia^34^, the Netherlands^41^, Spain^17^, France^12^ as well as in a sample of adolescents from China^40^, Iran^42^, the United Kingdom^43^ and university students from Brazil^44^. The measurement invariance of FAS scores across gender groups reached support in most translation test adaptation studies^45^. Some studies revealed that women have higher FAS scores compared to men^39,41^ and others reported the opposite^34,42^. Nevertheless, a recent meta-analysis revealed no significant differences in FAS between men and women^11^. Finally, Todd and Swami^37^ demonstrated that FAS scores are partially invariant across adults from the United Kingdom and Malaysia, with adults from the United Kingdom reaching significantly lower FAS scores.

Based on a global demonstration of the FAS’s good psychometric properties, it is predictable that the instrument will be increasingly employed by researchers aiming not only to holistically assess PBI but also to use it in various interventions. Also, it is important to continue the exploration of the FAS in various cultures and countries with different linguistic contexts. Although the psychometric properties of the Polish version of the FAS have been examined^38^, to the best of our knowledge, the applicability of the instrument has been limited to Baltic countries including Lithuania. The Baltic countries have undergone rapid social and cultural change since their independence and, like Western countries, are now experiencing socio-cultural factors that influence their populations’ lifestyles and body image^46^. In Lithuania, there are lack of measures assessing the multiple aspects of PBI, and, to date, no instrument is available to assess body functionality appreciation. Measuring facets of PBI such as FAS and promoting PBI and specifically body functionality appreciation is an important scientific and practical task; thus, internationally sound measures should be validated for this purpose.

To fill this gap and contribute to the body of ongoing cross-national research, this study sought to examine the psychometric properties of the Lithuanian translation of the FAS in a sample of young adults. To achieve this aim, the following objectives were set: (1) to identify the appropriate factor structure; (2) to examine gender invariance at the configural, metric, scalar and strict levels and to compare mean scores between genders; (3) to assess the composite reliability of the received model; and (4) to investigate the broader indices of construct and incremental validity.

Initially, following test adaptation recommendations^47^ and suggestions for body image instrument adaptation^48^, we planned to use the exploratory to confirmatory factor analytic method (EFA to CFA) to identify the appropriate factor structure of FAS scores. Following previous studies, we expected that FAS scores would replicate the original 1-dimensional FAS model. Consistent with previous studies^10,13–15^, we expected that the 1-dimensional FAS model would support configural, metric, scalar and strict invariances across gender groups. Achieving the invariance of the FAS structure would also allow us to compare mean scores of FAS between genders. We hypothesized that no significant differences would be observed between genders.

Additionally, here we also sought to assess the composite reliability of the received model. To assess construct validity, we selected measures that have been validated for use in Lithuanian and that have been reported in previous studies to be significantly associated with functionality appreciation^10,14,39^. Specifically, we expected convergent validity to be supported by a positive association between FAS, body appreciation, and physical activity habits. We expected to find support for convergent validity through a negative association between FAS and disordered eating and BMI. To assess criterion-related validity, we expected to confirm a significant positive correlation between the FAS and self-esteem. Finally, we hypothesised that FAS scores would predict unique variance in self-esteem over and above associations with body appreciation, symptoms of disordered eating, BMI and physical activity habits.

## Methods

### Participants

A total of 759 adults participated in the study. The mean age of the study participants was 23.2 ± 7.1 years (age range 18–44 years). Of all the participants, 335 (44.1%) were men and 424 (55.9%) were women. The body mass index (BMI) ranged from 14.9 to 44.8 (M = 23.2, SD = 3.8) kg/m^2^. The majority of participants were classified as having body weight in the healthy range (68.4%), 19.9% were overweight, 5.0% were obese, and 6.7% were underweight.

### Procedures

Data was collected between November 2022 and May 2023 via the Survey Monkey Research Panel (www.surveymonkey.com). The duration of completion of the survey was 20–25 min. When the participants agreed to participate in the survey, they were asked to provide digital informed consent. Respondents who provided informed consent were directed at the measures described below in the Measures Section. If a participant refused to participate in the study, the survey was terminated. The participants were provided with the opportunity to decline participation at any point by closing the browser without any recorded answers.

Data was collected in the universities of Lithuania. Upon receiving permission from the administration staff of the university, the trained researcher (V.B.) provided the survey link to the potential participants in their classrooms before or after lectures with no time limit for its completion. Participants did not receive any financial or other reward for participation in the study.

### Test adaptation

Following the guidelines for the instrument’s translation and recommendations for the test adaptation of body image instruments, the original FAS was translated into Lithuanian^48^. Our procedure consisted of an initial translation of the original FAS by two independent bilingual translators, a synthesis of the translations by resolving any discrepancies according to the translators’ reports, a back-translation by two independent bilingual translators, and an evaluation of the initial and back-translations by experts and scientists working in the field. No major translation problems were identified and the committee of experts and scientists approved the final version of the FAS.

### Measures

#### Demographics

Participants provided demographic information including gender and age.

#### Functionality appreciation scale (FAS)

The Lithuanian translated version (FAS) of the FAS original^10^ was used in the present study. The original FAS consists of seven items rated on a 5-point Likert scale ranging from 1 (strongly disagree) to 5 (strongly agree).

#### Body appreciation scale 2 (BAS-2)

The Body Appreciation Scale-2 (BAS-2^49^; Lithuanian translation^50^ was used to assess body appreciation. The unidimensional instrument comprises 10 items that are rated on a 5-point Likert scale, with the possibility to answer from “Never” (1) up to “Always” (5). Item examples are “I respect my body”; “I appreciate the different and unique characteristics of my body”. An overall score on the scale was calculated by averaging all items and a higher score indicated greater body appreciation. In a previous study, the BAS-2 demonstrated a unidimensional factor structure and good psychometric properties in a sample of Lithuanian youths^50^. In the present sample, the composite reliability (McDonald’s ω) was good in men and women groups: 0.95 (95% CI = 0.94, 0.95) and 0.97 (95% CI = 0.96, 0.97), respectively.

#### Rosenberg Self-esteem scale (RSES)

The Rosenberg Self-Esteem Scale (RSES^51^; Lithuanian translation^52^ was applied to assess self-esteem. The self-report instrument consists of 10 items, with five of the items positively worded (i.e. “I feel that I have a number of good qualities”) and the other five negatively worded (i.e. “I feel I do not have much to be proud of”). The items were scored on a 4-point Likert scale, ranging from 1 (strongly disagree) to 4 (strongly agree), yielding scores from 10 to 40. Higher scores indicate higher self-esteem. The RSES has shown factorial validity^52^ and has therefore been widely used in Lithuanian populations. In the present study, the composite reliability (McDonald’s ω) was good in men 0.87 (95% CI = 0.85, 0.89) and women 0.91 (95% CI = 0.90, 0.92).

#### Eating disorder examination questionnaire 6 (EDE-Q 6)

The Eating Disorder Examination Questionnaire-6 (EDE-Q 6^53^; Lithuanian translation^54^ was used to assess the characteristics of disordered eating behaviors and/or eating disorders during the previous 28 days. The instrument comprises 28 items, of which six are open-ended questions (for assessment of binge eating, self-induced vomiting, laxative use, and excessive exercise) and 22 are attitudinal questions from four subscales (for assessment of restraint, eating, shape, and weight concerns). Item examples of attitudinal questions are: “Have you had a definite desire to have an empty stomach with the aim of influencing your shape or weight?” or “Over the past 28 days, on how many days have you eaten in secret (i.e. furtively)”. The answer options are arranged on a 7-point Likert scale, with the possibility to answer from “No days,” “Not once,” or “Not at all” (0) up to “Every day,” “Every time,” and “Markedly” (6). Subscale scores were calculated as the mean of all items, with higher scores indicating greater disordered eating symptomatology. We applied only the overall EDE-Q 6 score in the present study. A previous study showed that the EDE-Q 6 has adequate patterns of acceptable psychometric characteristics in a sample of Lithuanian university students^54^. In the present study, the composite reliability with McDonald’s assessment for the EDE-Q-6 was 0.93 (95% CI = 0.91, 0.94) in men and 0.94 (95% CI = 0.94, 0.95) in women.

#### Self-Report habit index (SRHI)

The Self-Report Habit Index (SRHI;^55^; Lithuanian translation^56^ was used to measure the strength of physical activity habits. The unidimensional instrument is designed to evaluate different habitual behaviors, such as sedentary behavior, physical activity, and nutrition. In this study, the SRHI was adapted for physical activity. The SRHI consists of a stem (‘Behaviour X is something…’) that is adapted for different behaviours (e.g. ‘Physical activity is something…’), followed by 12 items with Likert response options. The answer options range from completely in disagreement (1) to completely in agreement (7). A sample item is “…I do frequently”. Items are summed and averaged to get an overall SRHI score with a higher score reflecting higher strength of physical activity habits. Adequate psychometric properties and a 1-factor structure have been reported for the SRHI in a sample of the Lithuanian general population^56^. In the present study, the composite reliability with McDonald’s assessment for the SRHI was 0.93 (95% CI = 0.92, 0.94) in men and 0.95 (95% CI = 0.94, 0.96) in women.

#### Body mass index (BMI)

Participants were required to self-report their height and weight to compute their self-reported BMI (kg/m^2^). BMI classification into categories was conducted according to the World Health Organization’s recommendations^57^. Accordingly, four groups of BMI were identified: underweight (< 18.5 kg/m^2^); normal weight (18.5–24.9 kg/m^2^); overweight (25.0–29.9 kg/m^2^); and obese (≥ 30.0 kg/m^2^).

### Analytic strategy

#### Data treatment

Statistical analyses were conducted with the SPSS v.29 and JASP v.0.19.1. Confirmatory factor analysis and invariance testing between gender groups were conducted with CFA using the *Lavaan*, *semTools*, and *MVN* packages with R v.4.4.2^58^. As the online survey required responses to all items to complete the survey, there were no missing values in the dataset.

Preliminary analysis included descriptive statistics and normality testing of the study variables. We randomly split the sample by IBM SPSS random sample function resulting in one split-half for EFA (*n* = 379; women *n* = 212, men *n* = 167) and a second split-half for CFA (*n* = 380; women *n* = 212, men *n* = 168). No significant differences were found between the two split-half samples in terms of gender groups (χ² = 0.002, df = 1, *p* = .967), mean age (sample 1: M = 22.94, SD = 7.36, sample 2: M = 23.39, SD = 6.94, t(757) = − 0.87, *p* = .385, d = − 0.06), or mean BMI (sample 1: M = 23.09, SD = 3.54, sample 2: M = 23.31, SD = 3.97, t(757) = − 0.81, *p* = .417, d = − 0.06). To test the FAS factor structure, we used the EFA–CFA strategy as recommended by Swami, Todd and Barron^59^.

#### Exploratory factor analysis (EFA)

Considering the sample adequacy for the EFA, a general rule was applied: thumb is at least 5–10 participants per item, though larger samples improve stability. Data factorability was assessed using the Kaiser–Meyer–Olkin (KMO) measure of sampling adequacy and Bartlett’s test of sphericity. A KMO value closer to 1 (ideally ≥ 0.80) indicates a high degree of common variance among variables, suggesting that factor analysis is appropriate^60^. Principal Axis Factoring (PAF) was used for EFA to identify the underlying latent variables (factors) that explain the shared variance among observed variables while accounting for measurement error. PAF is robust to deviations from normality^61^. Factor loadings of ≥ 0.45 were retained^62^.

#### Confirmatory factor analysis (CFA)

CFA was conducted to establish the fit of the FAS to the hypothesized one-factor model developed by Alleva^10^. Monte Carlo simulations with varying seed values, utilizing the factor loadings reported by Alleva et al., have demonstrated that a sample size of roughly 180 is adequate for this analysis. The assumption of normality was not met (Skewness ranged between − 0.87 and − 1.27 in men and between − 1.10 and − 1.46 in women, Kurtosis between 0.55 and 2.10 and 1.35–2.88, respectively). Mardia’s test of multivariate normality in men was for Skewness 715.6, Kurtosis 31.1, *p* < .001, in women Skewness 1017.4, Kurtosis 41.1, *p* < .001. Thus, the CFA model was estimated using the robust maximum likelihood approach, with fit indices adjusted through the Satorra–Bentler (SB) correction^63^. The goodness of fit was tested using the Comparative Fit Index (CFI), the Tucker-Lewis Index (TLI), the Root Mean Square Error of Approximation (RMSEA) and the Standardized Root Mean Square Residual (SRMR). To assess goodness of fit, we used the model chi-square (χ^2^/df; values < 3.0, considered indicative of good fit), the RMSEA and its 90% CI (with values close to 0.06 considered to be indicative of good fit and up to 0.08 indicative of adequate fit), the SRMR(values < 0.09 indicative of good fit), the CFI (values close to or > 0.95 indicative of adequate fit), and the TLI(values close to or > 0.95 indicative of good fit)^64^.

#### Gender invariance

Ultimately, to ascertain whether the FAS remains consistent across gender groups, we evaluated measurement invariance. Initially, a configural invariance model was employed to examine if the underlying factor structure remained unchanged across both groups without imposing equality constraints. Subsequently, metric invariance was investigated by equalizing factor loadings across gender groups and comparing the configural model to the metric model. Lastly, scalar invariance was examined by enforcing equal intercepts across groups, and the scalar model was compared to the metric model. Changes in model fit indices such as CFI, RMSEA, and SRMR were analyzed according to the criteria outlined by Chen^65^ and Sass^66^: CFI < 0.01 and RMSEA < 0.015 or SRMR < 0.030.

#### Further analyses

The composite reliability of the study measures was tested by McDonald’s ω and presented with 95% confidence interval^67^. The ω of ≥ 0.70 was considered adequate composite reliability^68^. After scalar invariance across gender groups was established, an independent sample t-test was used to compare FAS mean scores between men and women.

To assess convergent and criterion-related validity, after the distribution normality check, the Pearson or Spearman correlation coefficients were employed to examine the bivariate correlations between FAS scores and scores on the other study measures included in the survey. Correlations between 0.1 and 0.3 were considered weak, while those above 0.3 and below 0.5 were considered moderate, and above 0.5 were considered strong, with the level of significance at < .05^69^.

The incremental validity of the FAS was assessed by determining whether its scores significantly predicted self-esteem. Separately, for men and women, hierarchical regression analyses were conducted where BMI, body appreciation, PA habits and symptoms of disordered eating were entered in the first step, and body functionality appreciation was added in the second step in the prediction of self-esteem. Evidence of incremental validity was indicated if a statistically significant increment in Adj. R^2^ at the second step in the regressions was obtained.

### Ethics approval

The study was conducted following the ethical and legal principles of the Declaration of Helsinki, and ethical approval was obtained from the Social Research Ethics Board of Lithuanian Sports University (protocol number SMTEK-131, October 30, 2022).

### Consent to participate

Informed consent was obtained from all participants of the study.

## Results

### The exploratory (EFA) and confirmatory (CFA) factor analyses and measurement invariance across gender groups

In men (*n* = 167), Bartlett’s test of sphericity, χ^2^(21) = 434.0, *p* < .001, and KMO (0.91) indicated that the FAS items had an adequate common variance for factor analysis. EFA revealed a single factor with an eigenvalue greater than 1 (λ = 4.67) which explained 63.3% of the common variance. Factor loadings from the EFA and Lithuanian translation of the FAS items are presented in Table 1. In women (*n* = 212) the KMO test was 0.92, and Bartlett’s test of sphericity χ^2^ = 1190.1, df = 21, *p* < .001. EFA again revealed a single factor with an eigenvalue greater than 1 (λ = 5.15), which explained 69.2% of the common variance.

In addition, composite reliability for the FAS was calculated for women (ω = 0.94, 95% CI = 0.93, 0.95) and men (ω = 0.91, 95% CI = 0.89, 0.93) in the same split-half subsample and were adequate.

**Table 1:** Functionality appreciation scale item factor loadings from exploratory factor analysis from the first split-half subsample for men and women (*n* = 379). Lithuanian translations are provided in italic font.

Items	EFA subsample (*n* = 379)	
	Men (*n* = 167)	Women (*n* = 212)
(1) I appreciate my body for what it is capable of doing/ *Aš vertinu savo kūną už tai*,* ką jis sugeba*	0.69	0.82
(2) I am grateful for the health of my body, even if it isn’t always as healthy as I would like it to be/ *Esu dėkingas/a už savo kūno sveikatą*,* net jei jis ne visada toks sveikas*,* kokio norėčiau*	0.73	0.84
(3) I appreciate that my body allows me to communicate and interact with others/ *Vertinu*,* kad mano kūnas leidžia man bendrauti ir sąveikauti su kitais*	0.83	0.84
(4) I acknowledge and appreciate when my body feels good and/or relaxed/ *Pripažįstu ir vertinu*,* kai mano kūnas jaučiasi gerai ir / arba atsipalaidavęs*	0.76	0.85
(5) I am grateful that my body enables me to engage in activities that I enjoy or find important/ *Esu dėkingas/a*,* kad mano kūnas leidžia man užsiimti veikla*,* kuri man patinka arba kuri man svarbi*	0.84	0.83
(6) I feel that my body does so much for me/ *Jaučiu*,* kad mano kūnas dėl manęs daro labai daug*	0.78	0.80
(7) I respect my body for the functions it performs/ *Gerbiu savo kūną už jo atliekamas funkcijas*	0.84	0.83

**Fig. 1:**
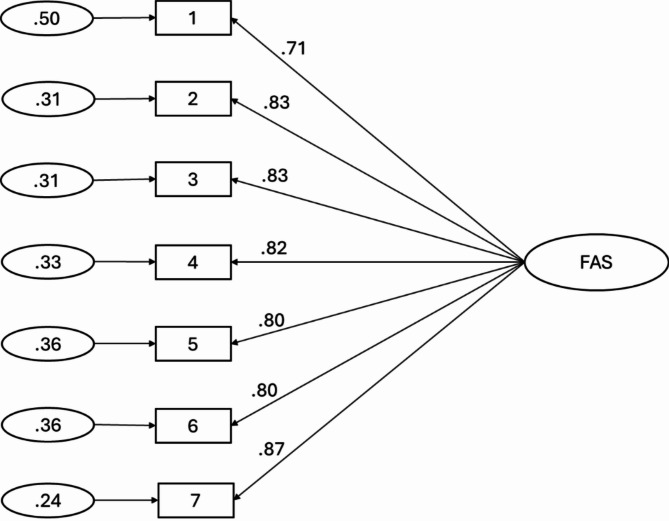
Path diagram and estimates for the 1-dimensional model of Functionality Appreciation Scale scores in the second split-half subsample. The large oval is the latent variable, with the rectangles representing measured variables, and the small ellipses representing the residual variances). The path factor loadings are standardised (all *p* < .001)..

Next, CFA of the FAS was conducted in the second split-half subsample and revealed an adequate fit to data indices for a unidimensional model supported by the EFA: SB χ²(14) = 28.74, robust RMSEA = 0.083 (90% CI = 0.038, 0.126), SRMR = 0.027, robust CFI = 0.980, robust TLI = 0.971. Standardized factor loadings with residual variances are presented in Fig. 1.

Next, we tested for measurement invariance across gender groups for the full subsample. Model comparisons did not indicate significant differences, and changes in model fit indices were minimal. As reported in Table 2, indices supported configural, metric, scalar and strict invariance. RMSEA values were slightly higher than the ideal range (< 0.08), but changes across models were minimal, supporting invariance. This allows meaningful comparisons of factor structure, latent constructs, and observed scores across genders.

**Table 2:** Measurement invariance of functionality appreciation scale across gender groups in the second split-half subsample (*n* = 380).

Model	SBχ^2^	df	Robust CFI	Robust RMSEA	SRMR	Model comparison	∆SBχ^2^	∆Robust CFI	∆Robust RMSEA	∆SRMR	∆df	*p*
Configural	58.0	28	0.966	0.110	0.032							
Metric	66.83	34	0.965	0.103	0.058	Configural vs. metric	8.83	0.001	0.007	0.026	6	0.243
Scalar	76.83	40	0.963	0.096	0.060	Metric vs. scalar	10.00	0.002	0.007	0.002	6	0.205
Strict	88.82	47	0.956	0.098	0.063	Scalar vs. strict	11.99	0.007	0.002	0.003	7	0.09

*SB*  Satorra-Bentler, *df* degrees of freedom, *CFI* comparative fit index, *RMSEA* Steiger–Lind root mean square error of approximation, *SRMR* standardized root mean square residual.

Finally, composite reliability for the FAS was calculated for women (ω = 0.94, 95% CI = 0.92, 0.95) and men (ω = 0.92, 95% CI = 0.90, 0.94) in the second split-half subsample and was also adequate. After calculating FAS mean scores, the comparison across men and women was conducted and no significant difference was found (men M = 4.17, SD = 0.73, women M = 4.26, SD = 0.75, t(377) = − 1.15, *p* = .251, d = − 0.12).

### Convergent and criterion-related validity

Table 3 represents the assessment of the validity of FAS scores, employing Pearson or Spearman correlation coefficients between FAS and study measures separately for men and women using the total sample. A significant, strong, and positive correlation was found between FAS and body appreciation in men and women. In both men and women, FAS was significantly and positively associated with the strength of physical activity habits. A significantly moderate negative correlation was found between FAS and disordered eating in women and men. Significant, positive, and moderate to strong associations between FAS and self-esteem were observed in men and women. Finally, in women, a significant, negative, and weak correlation was observed between FAS and BMI.

**Table 3:** Bivariate correlations between scores on other measures included in the study and the Lithuanian translation of the functionality appreciation scale (FAS) in men (*n* = 335, top diagonal) and women (*n* = 424, bottom diagonal).

Study measures	(1)	(2)	(3)	(4)	(5)	(6)
(1) FAS		0.73**	0.30**	– 0.30**	0.54**	– 0.05
(2) BAS-2	0.67**		0.30**	– 0.31**	0.65**	– 0.04
(3) SRHI	0.28**	0.28**		0.08	0.15**	0.12*
(4) EDE-Q 6	– 0.30**	– 0.56**	– 0.01		– 0.40**	0.29**
(5) RSES	0.46**	0.64**	0.24**	– 0.47**		0.05
(6) BMI	– 0.15**	– 0.23**	– 0.09	0.29**	– 0.01	

*FAS* Functionality Appreciation Scale, *BAS-2* Body Appreciation Scale 2, *SRHI* Self-Report Habits Index, *EDE-Q 6* Eating Disorders Examination Questionnaire 6, *RSES* Rosenberg Self-Esteem Scale, *BMI* Body Mass Index.

*p < .05, **p < .001..

### Incremental validity

Separately, for men and women, hierarchical regressions were performed to test incremental validity. Hierarchical regression models were developed for the gender groups using the enter method. In models, self-esteem was considered a dependent variable, and BMI, body appreciation, PA habits and symptoms of disordered eating were regarded as independent variables in the first step. In the second step, body functionality appreciation was added as an independent variable. The results of hierarchical regression analyses for the prediction of self-esteem are provided in Table 4. In men, the first step of the regression was significant (F = 75.20, *p* < .001, Adj. R^2^ = 0.47), as was the second step (F = 62.80, *p* < .001, Adj. R^2^ = 0.48). In the second step, the addition of functionality appreciation accounted for a significant incremental change in Adj. R^2^ (ΔR^2^ = 0.012, *p* = .007). In women, the first step of the regression was significant (F = 91.72, *p* < .001, Adj. R^2^ = 0.46). The second step of the regression also demonstrated significance (F = 75.73, *p* < .001, Adj. R^2^ = 0.47). The addition of functionality appreciation accounted for a significant incremental change in Adj. R^2^ (ΔR^2^ = 0.008, *p* = .01). All variance inflation factors (VIFs) in both regression models were ≤ 2.69, suggesting that no multicollinearity problem was detected.

**Table 4:** Results of hierarchical regression analyses for the prediction of self-esteem (*n* = 759).

Step	Study variables	Men (*n* = 335)			Women (*n* = 424)		
		B	β	*p*	B	β	*p*
1	BMI	0.24	0.14	< 0.001	0.31	0.19	< 0.001
	BAS-2	3.95	0.59	< 0.001	3.70	0.54	< 0.001
	PA habit index	– 0.08	– 0.02	0.656	0.37	0.09	0.016
	EDE-Q 6	– 1.28	– 0.25	< 0.001	– 0.98	– 0.21	< 0.001
	*Model parameters*:	*R* = .69; R^2^ = 0.48; Adj. R^2^ = 0.47			*R* = .68; R^2^ = 0.47; Adj. R^2^ = 0.46		
	F	75.20			91.72		
	p	< 0.001			< 0.001		
2	BMI	0.24	0.14	< 0.001	0.31	0.19	< 0.001
	BAS-2	3.26	0.48	< 0.001	3.04	0.45	< 0.001
	PA habit index	– 0.17	– 0.04	0.330	0.32	0.08	0.039
	EDE-Q 6	– 1.21	– 0.24	< 0.001	– 1.07	– 0.23	< 0.001
	FAS	1.30	0.16	0.007	1.10	0.13	0.010
	*Model parameters*:	*R* = .70; R^2^ = 0.49; Adj. R^2^ = 0.48 ∆*R* = .12, ∆F = 7.50, p ∆F = 0.007			*R* = .69; R^2^ = 0.48; Adj. R^2^ = 0.47 ∆*R* = .008, ∆F = 6.74, p ∆F = 0.010		
	F	62.80			75.73		
	p	< 0.001			< 0.001		

*B* unstandardized regression coefficient, *β* standardized regression coefficient, *p* level of statistical significance, *BMI* body mass index, *BAS-2* Body Appreciation Scale 2, *PA* physical activity, *EDE-Q 6*  Eating Disorders Examination Questionnaire 6, *FAS* Functionality Appreciation Scale.

## Discussion

The FAS, developed to measure appreciating, respecting, and honouring the body for what it is capable of doing, has been shown to have good psychometric properties in samples of a diverse range of national and linguistic contexts. However, its psychometric properties have been not tested in samples of Baltic countries, including Lithuania. Thus, in the present study, we aimed to fill this gap. The results of the present work supported the unidimensional model of the FAS using EFA and CFA, with all seven items retained in the final model. Factor loadings were high suggesting that the factor structure of the scale is robust. Moreover, we found that FAS demonstrated invariance across genders, and scores of the instrument showed adequate composite reliability and adequate convergent, concurrent, and increment validity. Overall, these findings suggest that the Lithuanian translation of the FAS has good psychometric properties. Further, we discuss these results more specifically.

In terms of factorial validity of the Lithuanian translation of the FAS, our results follow previous work showing that the scale is unidimensional and replicates the original measure^10^. The unidimensional structure of the scale was confirmed in other samples of different national and linguistic contexts^13–16,19,34,39,41,42^. This finding suggests that the FAS is a stable measure that assesses latent functionality appreciation in a diverse cultural context and might be a useful tool for determining functionality appreciation across nations, including Eastern Europe.

The results of the present study showed that the unidimensional factor structure of the FAS was identical across women and men and achieved invariance across gender groups. This suggests that the instrument measures the same latent construct of functionality appreciation in both women and men. We also found that the mean FAS scores were similar to those in some Western countries^14,17^. No differences in mean scores between women and men were observed. These results are consistent with previous evidence^13–17^. Our results suggest that the Lithuanian cultural context may not have a significantly different impact on the FAS compared to the context of Western countries.

The results of the present study also showed that Lithuanian FAS had adequate composite reliability, with McDonalds‘ ω coefficients consistently exceeding a cut-off score of .70^70^. Generally, our results support the construct validity of the Lithuanian FAS, since the scores of FAS significantly correlated with the scores for body appreciation and physical activity habits. Previous studies also showed that FAS positively correlated with body appreciation which supports the instruments’ concurrent validity^13–17,34,39,41^. Results of the previous studies showed that physical activity has the potential to focus individuals‘ attention toward their body functionality, and foster functionality appreciation^71^. Alternatively, people who can appreciate the functionality of the body may invest more in body-related practices such as physical activity^27,72^.

In line with previous studies^11^, FAS scores were weakly but significantly negatively correlated with scores of disordered eating and BMI (in women), which supports the measure‘s construct discriminant validity. Previous FAS validation studies also reported negative FAS association with disordered eating^13–15,17,39^. However, we found no correlation between BMI and functionality appreciation in men. Similar results in men were observed in some other studies^15,34^ while other part of studies reported significant negative associations between FAS and BMI in men^13,17^. These results might be explained by the differences in the age and the distribution of overweight respondents between samples.

Further, our results provide support for the incremental validity of FAS. The FAS uniquely predicted self-esteem after accounting for the variance explained by other measures, such as BMI, body appreciation, disordered eating and physical activity habits. In the second step, the functionality appreciation accounted for a small incremental percentage of the variance explained; however, this translated to a significant effect in terms of incremental validity^73^. This finding is in line with the previous work^10,14,15,39^.

Several important limitations of the present study must be acknowledged. First, our sample does not represent all Lithuanian population, thus generalization of the results is limited. Further, the sample consisted of young university-aged people of two genders. In future studies, it is important to investigate the translated FAS among groups with different ages, gender identities, socioeconomic backgrounds, and eating habits, as well as samples with different physical health statuses and mental health conditions. The next limitation of the present study is that we did not assess test-retest reliability. Previous studies reported that FAS scores remained stable for several weeks^13,10^, however in future work, the test-retest reliability of the Lithuanian version of the FAS must be tested. Finally, an important consideration is whether the Functionality Appreciation Scale (FAS) items are too simplistic for community adult populations. A previous Rasch analysis suggested that incorporating more complex items could improve its suitability for community samples^74^. Thus, it is important to consider this issue in future studies.

Despite these limitations, the present study provides evidence that the Lithuanian FAS is a psychometrically valid measure that might be used to test functionality appreciation in adults. The results of the present study add to the growing body of evidence indicating that the FAS is a reliable and valid instrument that might be used in various countries with different nationalities and linguistic contexts^13–16,19,37–40,42–44,75–80^. The availability of the nationally validated FAS would facilitate future scholarly work on positive body image in Lithuania. The inclusion of functionality appreciation in intervention programs will also help to gain a deeper understanding of the importance of positive body image and body functionality for a healthy lifestyle, including mindful eating and physical activity.

## Electronic supplementary material

Below is the link to the electronic supplementary material.

Supplementary Material 1

## Data Availability

Data is provided within the supplementary information files. For study data requests, please contact the corresponding author, Migle Baceviciene.
